# Is Comprehension in Comics More Effective Than in Traditional Texts in Skilled Adult Readers? An Eye Movement‐Based Study

**DOI:** 10.1111/cogs.70081

**Published:** 2025-07-11

**Authors:** Maud Rasamimanana, Raphaël Mizzi, Jean‐Baptiste Melmi, Sophie Saffi, Pascale Colé

**Affiliations:** ^1^ Department of Cognitive Psychology, Aix‐Marseille University, CNRS, CRPN, InCIAM; ^2^ Aix‐Marseille University, CNRS, CRPN; ^3^ Department of Italian Studies, Aix‐Marseille University, CAER

**Keywords:** Reading comprehension, Comics, Eye movements, Skilled adult readers

## Abstract

Reading comprehension has been mostly studied using traditional texts and very little is known about reading comprehension in comics. We wanted to find out whether comics could enhance comprehension processes, compared to traditional text and what cognitive processes might be involved in this effect. Furthermore, we explored the functional role of pictures in understanding comics. Forty skilled readers read the comic and text versions of two already published stories and answered comprehension questions. Eye movements were recorded during reading. We found no differences in reading comprehension performance. However, comics were explored faster than traditional texts. Importantly, reading speed of words in balloons was faster than in traditional texts. An analysis of eye movements suggests that the presence of pictures facilitates the extraction of information, with shorter total saccadic amplitude on the pages of comics than in text. When reading comics, participants spent less time on the pictures than the balloons, and this behavior was associated with shorter and fewer fixations. Pictures were also used as an entry point for reading a panel, as the first fixation in the panel fell more frequently on the pictures and the readers returned to them more often than to the balloons. Because pictures are processed faster than words, they may be used to construct a first representation of the content of the story, which can be used to facilitate the processing of the whole story and, more specifically, of its verbal component. This strategy is not available in traditional texts.

## 1 Introduction

Comics constitute a representative example of multimedia formats—reading materials that integrate both text and images—and have gained substantial popularity in recent years, now being considered to be one of the most popular reading modes. For example, comic book sales in France increased by 55.8% between 2020 and 2021, making them the second largest sector of the publishing market (Syndicat National de l'Edition, 2022). In North America, comic book sales benefited from the outbreak of Coronavirus disease 2019 (COVID‐19), increasing by 62% during 2021, a trend which continued in 2022 (ICv2, 2023). Although the proportion of comic readers in the population decreases with age, comics “aren't just for kids,” as 43% of the French population between 16 and 75 years still read comics (Vincent Gerard, Chaniot, & Lapointe, 2020). Consequently, interest in comics and their potential use, in particular in the fields of literacy and education as well as for scientific communication, is growing fast (Crawford, 2004; Farinella, 2018; Jee & Anggoro, 2012; McVicker, 2007; Schwarz, 2006; Tatalovic, 2009).

Research on visual narratives has seen tremendous growth these last few years, and some studies have investigated the extent to which comprehension differs between textual and visual narratives. Several studies have compared film to written text (Baggett, 1979; Kendeou, Bohn‐Gettler, White, & van den Broek, 2008; Pezdek, Lehrer, & Simon, 1984; Zacks, Speer, & Reynolds, 2009), while others have examined the comprehension of sequential picture stories in contrast to written narratives (Magliano, Kopp, McNerney, Radvansky, & Zacks, 2012). These studies generally suggest that while both modalities can support narrative comprehension, they may engage different cognitive processes and emphasize distinct aspects of the story (e.g., temporal sequencing in film vs. inferential reasoning in text). Notably, many of these investigations have relied on wordless visual narratives (Baggett, 1979; Magliano et al., 2012; Zacks et al., 2009), limiting the generalizability of their findings to media that integrate both verbal and visual elements. Importantly, none of these studies have specifically examined multimedia comics (hereafter, comics), which combine images, text, and often dynamic visual transitions. This gap highlights the relevance of the present study, which aims to compare narrative comprehension in comics and traditional text formats.

More precisely, our aim in the present study was to better understand some of the cognitive processes involved in comprehension when reading comics including words. We investigated how readers build a mental representation of the story's content, that is, a situation model (van Dijk & Kintsch, 1983), from the processing of pictures and words in balloons (Jee & Anggoro, 2012), compared to traditional texts. We also sought to examine the interplay between these two components, focusing on how pictures may influence the reading of words in comics in contrast to traditional texts.

### 1.1 How do we read/process comics?

Comic books are a particular form of multimedia format that mobilize both words and pictures to narrate a story or convey information (Jee & Anggoro, 2012). As such, comics have two specific characteristics that are not found in other multimedia texts (e.g., textbooks or newspapers). First, in most multimedia formats, pictures and text are juxtaposed next to each other, while the verbal and pictorial components in comics are fully integrated, as texts are embedded in the pictures by means of balloons and captions. The spatial proximity of pictures and text may promote the integration of the two components, which could in turn foster reading and learning (Johnson & Mayer, 2012), especially in poor readers (Yum, Cohn, & Lau, 2021). Second, multimedia formats often include only one picture, while comics present multiple pictures on the same page or in the same layout. These pictures are organized in a meaningful sequence (Cohn, Paczynski, Jackendoff, Holcomb, & Kuperberg, 2012; Foulsham, Wybrow, & Cohn, 2016; McCloud, 1993). Thus, the cognitive processes involved in the integration of the two modalities (pictorial and verbal) in comics may differ from those deployed in response to multimedia formats that only contain one picture. Indeed, in multimedia formats, the pictorial component only plays a supportive semantic role and may be removed with little effect on comprehension. In comics, the pictorial and the verbal components are both necessary for comprehension (Cohn, 2016; Cohn & Schilperoord, 2022), although they may carry different informational weight (McCloud, 1993, 2006). For all these reasons, the term “multimedia formats” will be used in this article to refer to “classical” multimedia texts.

To account for the cognitive mechanisms involved in visual narrative comprehension, several models have been proposed. The Scene Perception and Event Comprehension Theory (SPECT; Loschky et al., 2020) emphasizes the interplay between attentional selection, information extraction, and event model construction during the viewing of visual narratives. The Parallel Interfacing Narrative‐Semantics (PINS) model (Cohn, 2020b) posits that semantic content and narrative structure are processed in parallel, allowing readers to build a situation model while assigning narrative roles to images. The Multimodal Parallel Architecture (MPA; Cohn, 2016) provides a broader framework for understanding how verbal, visual, and bodily modalities interact, each with their own form, meaning, and grammar. However, these models do not fully account for how readers integrate pictures and embedded text in static visual narratives such as comics. SPECT is tailored to dynamic media and does not address the influence of visual context on verbal processing. PINS focuses on narrative grammar within image sequences but overlooks the modulation of verbal comprehension by images. MPA offers a structural taxonomy of multimodal interactions but does not specify how pictures and words are jointly processed in real time. As such, these frameworks fall short in explaining how visual context affects the construction of meaning from written language in comics, particularly in comparison with traditional texts.

Multimedia formats have been shown to be an effective way to convey information, as learning from combined text and image is more efficient than learning from text without pictures (see for reviews: Anglin, Vaez, & Cunningham, 2013; Houts, Doak, Doak, & Loscalzo, 2006; Levie & Lentz, 1982; Mayer, 2009). This effect is known as the *multimedia effect*. Mayer (2009) proposed an interesting general framework, named the cognitive theory of multimedia learning (CTML), to explain the benefits multimedia texts may have compared to text alone. According to this model, reading and learning from multimedia formats require three different processing steps. First, attentional processes are deployed to extract, that is, *select*, relevant words and pictures. Next, the selected information is *organized* into two coherent models, resulting in the generation of one pictorial and one verbal model. Finally, the two models are *integrated* into a unified final model, allowing readers to fully make sense of the content of the support. Thus, adding pictures to a text would stimulate readers to create a pictorial and a verbal model of the content, allowing them to build connections between these two models, which in turn could enhance comprehension and learning.

Sonine and Chanquoy's (2007) model is another proposal that focuses specifically on comic comprehension processes. In particular, this model builds on the numerous results reporting that semantic access is faster for pictures than for words (see, e.g., Bajo, 1988; Potter & Faulconer, 1975; Glaser, 1992, for a review). In this model, the pictorial and verbal components of comics are processed in parallel but asynchronously, each following distinct temporal dynamics and cognitive pathways. First, the visual processing of both pictorial and verbal (word‐based) features is engaged in parallel. Readers then build mental representations of the verbal and the pictorial components. Semantic processing (i.e., the generation of the mental representation of the content) is faster for pictures than for the verbal component, as the former is not mediated by words. As a result, readers are able to elaborate an initial representation of the comic's content, named the basic representation, based on the content of the pictorial representation. This basic representation may stimulate mechanisms that either suppress or enhance potential concurrent semantic interpretations and thereby facilitate the ongoing semantic processing of the verbal component as suggested by the Structure Building Model (Gernsbacher, Varner, & Faust, 1990). Thus, in this model, picture processing may preactivate relevant semantic information, which is then used in a top‐down manner for processing the content of balloons and captions, thereby facilitating the processing of the verbal component. This hypothesis is supported by later studies that investigated whether viewing a picture before reading a text may increase reading speed (Eitel, Scheiter, & Schüler, 2013; Lindner, Eitel, Strobel, & Köller, 2017). The final step in Sonine and Chanquoy's (2007) model consists of integrating the verbal representation with the basic representation, which is done by reinforcing or updating this first semantic representation. Thus, the processing of comics may result from an “identification–processing–interaction” cycle, which may be repeated for each fragment/panel of the comic.

To test their model, Sonine and Chanquoy (2007) presented comic strips panel‐by‐panel to adult skilled readers. They used three types of presentation: verbal component only, pictorial component only, or both components. Participants were asked to summarize each panel with one word. The results showed that the words elicited in the presentation of the verbal component‐only condition were more varied and less consistent than for the pictorial component only or for the whole, two‐component panel. Thus, the pictorial component activated a more strongly associated and narrower lexical and conceptual network. In addition, the overlap between the concepts elicited by the whole panel and the pictorial component was greater than that between the concepts elicited by the whole panel and the verbal component, suggesting that the representation of the comic strips is driven by the processing of the pictures.

A comparison of comics and traditional texts in L2 learners shows that comics exert a multimedia effect (Liu, 2004; Wong, Miao, Cheng, & Yip, 2017; Yum et al., 2021). However, the behavioral results for memory and comprehension performances in comics are inconsistent in skilled readers and L1 speakers. Previous results have shown better verbatim recognition (i.e., recognition of words or sentences displayed in the supports) in comics than in text (Short, Randolph‐Seng, & McKenny, 2013), together with better comprehension for literal questions (Aleixo & Sumner, 2017). However, several studies have reported no improvement on when answering literal (Lin, Lin, Lee, & Yore, 2014; Short et al., 2013; Yum et al., 2021) or inferential questions (Short et al., 2013). Finally, comics have been used to train decision‐making in expert U.S. Navy officers. Although training with comics or with texts did not result in differences in performances, comics were processed faster than texts, thus reducing the time needed for cognitive training (Nalu & Bliss, 2011).

Eye tracking has been widely used to explore reading processes in classical multimedia learning (Alemdag & Cagiltay, 2018) and could be of particular interest to study comics reading comprehension (Bateman, Beckmann, & Varela, 2018; Wada, 2023). Only a small number of studies of the reading of comics have used eye movements to better understand the cognitive processes involved in this activity. These studies show that pictures may play a central role in the comprehension of comics. The results of a study investigating the use by teenagers of comics combined with maps to learn geography showed that the more the participants looked at the pictorial components (maps and characters), the better their comprehension of the comics’ content was (von Reumont & Budke, 2020). Similarly, Laubrock, Hohenstein, and Kümmerer (2018) observed that adult skilled readers made more fixations on pictorial components than verbal components when returning to an already visited panel, probably because they tried to look for visual information that they had missed the first time. Finally, these studies using eye movements in comics suggest that pictures may prime lexical semantic processing inside a panel. Thus, in comics, Laubrock et al. (2018) observed that the first fixation on each panel landed more frequently on the picture than on the text (see also Carroll, Young, & Guertin, 1992, for similar conclusions in single‐panel comics). However, the organization of the panel, for example, in terms of the position and length of the text, appears to modulate the location of this first fixation on panels (Kirtley, Murray, Vaughan, & Tatler, 2018). Previous studies also found that comic expertise greatly influenced eye movements, leading to fewer regressions (Cohn, 2020a) and shorter fixations (Zhao & Mahrt, 2018).

### 1.2 The Present Study

Few previous studies have compared comprehension performances in comics and traditional texts, and the behavioral results are inconsistent. This could be the consequence of two main methodological choices. First, the method used to create the text and comic supports may have modified the content of the supports and may have resulted in the comprehension facilitation effect observed with comics in some studies. For example, in Aleixo and Sumner's (2017) study, texts were created from comics by removing the pictures and presenting the verbal components as continuous prose, with better comprehension being observed when both modalities were present. Conversely, creating comics on the basis of texts by adding them verbatim in the picture captions and balloons may reduce the benefit of the pictures, especially in skilled readers such as those participating in Yum et al. (2021). In most studies, comics have been on the basis of texts, and it is possible that the semantic contents may differ significantly between the two supports. For example, Lin et al. (2014) added everyday life context and humoristic contents to comics to enhance readers’ interest. More crucially, when using existing supports or creating new comics based on texts, the previously cited studies did not use any procedure to ensure that the two supports were equivalent in terms of transmitted semantic contents. Second, the presence of a time limit to read the comic or text, as in Short's (2013) study, could also benefit comprehension and memory for the comic supports as they may be read faster than traditional text.

To handle these methodological challenges, we used stories previously published in both formats and controlled for the provided semantic information. When adapting novels into comic form, authors make important choices about what and how verbal text is converted/translated into visual form (Saltzman, 2017) and how to arrange the panel to direct the reader's attention toward the information that is relevant for telling the story (McCloud, 2006). To take this particularity of comics into account, we performed a content analysis to ensure that both the text and comic supports presented all the information needed to understand the stories and answer subsequent questions. Finally, we recorded eye movements during reading. Although some research has studied the factors influencing eye movements when reading comics (Foulsham & Cohn, 2020; Ishii, Igaki, Kurata, Omori, & Masuda, 2004; Kirtley et al., 2018, 2023; Laubrock et al., 2018), no study has used eye movements to compare traditional texts and comics.

The present study aimed to answer three questions. The first was to determine whether it is easier to process the content of the story in comics than in text as suggested by the multimedia effect first described in the CTML (Mayer, 2009). In addition, Sonine and Chanquoy's (2007) model predicts that the presence of a picture could facilitate the processing of the verbal component. Thus, our second question concerned the processes involved in any facilitation effect we might observe. More precisely, we wanted to know how the presence of pictures in comics may impact the extraction of information from the page as a whole as well as from its verbal component. Finally, our last question aimed at clarifying the role of the pictures in the elaboration of the final representation of the content of comics.

#### 1.2.1 Do comics facilitate the elaboration of the representation of the content of the story?

Mayer's (2009) CTML proposed that the presence of pictures in comics can support the elaboration of the final semantic representation of the story. However, we are also aware that some previous studies have reported similar comprehension performance in skilled adult readers (Lin et al., 2014; Nalu & Bliss, 2011; Short et al., 2013; Yum et al., 2021) irrespective of whether they read comics or traditional texts and that better comprehension scores in comics may be associated with methodological bias. We therefore developed two alternative hypotheses that we could test based on the comprehension scores (literal and inferential questions about the story) for the whole story presented in the two formats. If the presence of pictures in comics can contribute to the elaboration of the final semantic representation, as predicted by Mayer's (2009) CTML, we should observe better performance on both literal and inferential questions after reading comics rather than texts. Otherwise, we may observe similar comprehension performance for both comics and traditional texts.

We also expected overall story exploration indicators such as total exploration time (i.e., the time needed to process the whole story, pictures, and balloons included in the case of comics) to be shorter in comics than in text because some of the information present in traditional texts is contained in pictures in comics and pictures are processed faster than text (Potter & Faulconer, 1975).

Finally, comics may also have an impact on the elaboration of the mental representation of the story's content via a facilitation of the processing of words in balloons. Sonine and Chanquoy's (2007) model predicts that the presence of pictures will facilitate the processing of the verbal component in comics, compared with traditional texts. We therefore tried to obtain a more precise measure of the efficiency of elaboration of the verbal representation based on comics, compared with text by calculating reading speed, that is, the number of words read in a minute (obtained by dividing the number of words in each support by the time spent reading in comic balloons or in traditional texts). Because the processing of pictures in comics may facilitate the processing of words in balloons, we expected reading speed to be faster in comics than in text (Eitel, Scheiter, Schüler, Nyström, & Holmqvist, 2013).

#### 1.2.2 How do comics facilitate comprehension processes?

To answer this question, we investigated two complementary hypotheses, both derived from Sonine and Chanquoy's (2007) model. First, this model suggests that the pictorial representation should be easier to elaborate than the verbal representation. To test this assumption, we analyzed the way pictures and balloons were explored and processed in comics with the help of indicators such as the viewing time, that is, time spent inside each type of area of interest (balloon vs. pictures), and the number and mean fixation duration in comics. We expected shorter viewing time, shorter mean fixation duration, and fewer fixations for pictures than for balloons. Second, as some information from traditional text is translated into pictures in comics, information may be extracted more efficiently from comic pages than from traditional texts. In other words, it would be easier to extract the semantic representation of the story that is being read in a comic than in a traditional text. The result could be that the participants’ gaze covers a shorter distance in a comic than in a traditional text (Kotval & Goldberg, 1998). As far as the verbal component is concerned, the mean fixation duration and the number of fixations are known to be linked to the difficulty of the text (Rayner, Chace, Slattery, & Ashby, 2006). Because the processing of words in comic balloons is expected to be facilitated by the concurrent processing of pictures, we expected there to be fewer and shorter fixations on the verbal components of comics than on texts.

#### 1.2.3 How do we integrate the pictorial and verbal components of comics?

Sonine and Chanquoy (2007) postulate that pictorial information is given priority when building the final representation of the contents of a comic book. In accordance with this hypothesis, fixations when entering a panel for the first time should initially land on pictures more often than during later fixations to the same panels (Laubrock et al., 2018). In addition, if the pictorial representation is used as a basis for integrating the two components, pictures may also be used for the integration of the verbal with the pictorial representation as well as for revising this basic representation. Thus, readers may need to look back at previously seen pictures, leading to a greater number of runs (i.e., sets of consecutive fixations in the same area of interest) in pictures than in balloons.

## 2 Materials and methods

### 2.1 Participants

A total of 40 participants (23 women and 17 men; mean age: 21.57), all students from Aix‐Marseille University (mean years of higher education: 2.8) and native French speakers, took part in the study. They had normal or corrected‐to‐normal vision, normal audition, and a non‐verbal IQ (Raven's matrices: Raven, 2003) and visuo‐spatial abilities (assessed with Complex Rey Figure; Rey, 1959) within the normal range. None of them reported either any neurological or psychiatric disorder.

We ensured that the participants had no history of reading difficulties (Adult Reading History Questionary under 0.47; Cavalli et al., 2024) and no reading impairments, all of them achieving a reading fluency score (CTL score) above the optimal cutoff of the French “Alouette” test (Cavalli et al., 2018). We assessed comic reading expertise with the Visual Language Fluency Index (VLFI, Cohn, 2014), as this can greatly influence the comprehension of comics. This index was computed for each participant based on the participant's comic reading and production habits, for different types of comics and during different periods of life. According to Cohn, most of the participants had low to average visual language fluency (low: *n* = 15, average: *n* = 22, high: *n* = 3; mean score: 10.86 and *SD*: 5.41, Cohn, 2014), suggesting a generally low familiarity with comic supports. Consequently, we did not find any correlation either between VLFI and comprehension scores for comics (*r* = 0.05, *p* = .074) or between VLFI and exploration time (*r* = −0.23, *p* = .15).

Written consent was obtained before the experiment. The research was approved by Aix‐Marseille University's Ethics Committee (no. 2021‐02‐11‐010).

### 2.2 Materials

Two stories published as traditional novels and adapted into graphic form were used in the experiment (T1 and T2). T1, entitled “C'est pour ça que je m'appelle Giovanni” (“That's why I'm called Giovanni”), was written by Luigi Garlando and adapted by Claudio Stassi. T2, entitled “Le Der des ders” (“The Last of the Last,” our translation), was written by Didier Daeninckx and illustrated by Jacques Tardi. T1 illustrations were in color, whereas T2 illustrations were in black and white. We selected the first pages of each support (excluding covers) to make sure that the readers had all the information needed to understand their content. The characteristics of the two stories are described in Table 1. The comics contained approximately the same number of panels (21 and 22 panels) and pages. In addition, the number of words in the comics amounted to approximately one‐third of the number of words in the original (33% for T1 and 37% for T2). However, T1 text version had fewer words and pages than T2.

**Table 1:** Characteristics of the novel and comic versions of the two stories

Title	C'est pour cela que je m'appelle Giovanni		Le Der des ders	
Medium	Novel	Comic book	Novel	Comic book
Author/writer	L. Garlando	C. Stassi	D. Daeninckx	D. Daeninckx
Artist		C. Stassi		J. Tardi
Year of publication	2004	2011	1988	1997
Number of pages	4	4	6	4
Number of panels		22		21
Number of captions and speech balloons		35		28
Number of words	1054	348	1394	522

The two stories were selected because their comic adaptions were similar to the original novels. To ensure that the two supports contained similar information, a linguist, specialist in comic corpora of our research team, constructed comparative tables for each story. These tables summarize all the semantic information present in the first pages of the novel, and the locations where this information is transposed to the comics, that is, in pictures, balloons, or captions.

Based on these tables, we created comprehension questionnaires for each story. These contained both literal questions, related to information explicitly present in both the comics and novels, and inferential questions, which required the participants to draw on their knowledge or to make connections between different pieces of information in the story. Questions could be open or multiple‐choice. The maximum score for literal questions was 8 for T1 and 11 for T2. The maximum score for inferential questions was 10 for both stories. Cronbach's alpha value was .705 across the two questionnaires and both types of questions taken together.

### 2.3 Procedure

Participants read both stories on counterbalanced media, with the first half of the group reading the comic version of T1 and the text version of T2, and vice versa for the other half. The order of media and stories was counterbalanced using a Latin square design. The participants sat at a comfortable distance (around 60 cm) from a portrait‐format screen that is suitable for comic‐book layouts. Page size was around 40 cm high. They could control their reading speed by moving from page to page using a keyboard in front of them but were not allowed to return to a previous page. After each story, they had to answer oral comprehension questions.

After the main comprehension task, the participants performed various control tasks designed to evaluate the different cognitive abilities described above.

### 2.4 Eye movements

Eye movements were recorded using an Eyelink Portable Duo (SR Research) in Head Free‐to‐Move Tracking mode. Head movements were limited by means of a chin rest. Gaze locations were recorded at a sampling rate of 1000 Hz, based on pupil and corneal reflection. Before each story was read, we performed a 9‐point calibration and validation, and this procedure was repeated until the validation error was smaller than 1° on average and 1.5° for the worst point. The mean validation error across participants was 0.35° (*SD* = 0.10). Drift correction was also performed between each page. The experiment was monitored with SR Research's Experiment Builder software.

Ocular data were extracted using Data Viewer's (SR Research) default parser. Because drift correction was centered on the page, the first fixation and saccade of each page were excluded from the analysis. After removing this first fixation, the first five fixations consistently landed within the first three panels of the page. Interestingly, only 56.9% of the first fixations fell within the first panel, while between 83% and 90% of the subsequent fixations were concentrated in the first panel (see Table 2).

**Table 2:** Proportions of the landing position of the first five fixations

	Fixation Number				
Panel Number	1	2	3	4	5
1	56.88	85.00	90.00	85.63	83.13
2	27.50	10.63	8.75	13.13	15.00
3	15.63	4.38	1.25	1.25	1.88

In the case of the comics, when a fixation did not fall within a specific area of interest (picture or text), we considered that it fell within the closest area of interest (based on the shortest distance to the closest edge of an area of interest). Around 5% of comic fixations were concerned.

## 3 Results

The data were processed and graphics produced using R language (R Core Team, 2022) and the Tidyverse package (Wickham et al., 2019). Statistical analyses were conducted using the rstatix (Kassambara, 2023), BayesFactor (Morey & Rouder, 2023), and JSmediation (Batailler, Muller, Yzerbyt, & Judd, 2023) packages.

### 3.1 Do comics facilitate the elaboration of the representation of the content of the story?

To answer this first question, we performed a repeated‐measures anova, with Media (comic vs. text) and Question Type (literal vs. inferential) as within variables for the **comprehension scores** (percentage of correct responses). We also calculated the **exploration time** determined as the time needed to process the whole story, pictures, and balloons included in the case of comics, and the **reading speed** determined as the number of words divided by the total reading time (the sum of all fixation and saccade durations for text supports and that of fixation and saccade durations directed toward balloons and captions for comics). We performed repeated‐measures anovas for these two dependent variables (1b and 1c) with Media as the within variable.

The descriptive statistics (means and standard deviations) are shown in Table 3.

**Table 3:** Descriptive statistics and *p*‐values associated with different measures comparing comics and texts

	Comics	Texts	*p*‐value
	Mean (*SD*)	Mean (*SD*)	
**Comprehension scores**	43.90 (25.47)	42.49 (22.04)	.755
*Literal Questions (max = 12)*	53.81 (26.44)	53.23 (20.91)	
*Inferential Questions (max = 12)*	34.0 (20.36)	31.75 (17.53)	
**Exploration time (s)**	156.93 (61.01)	340.43 (115.63)	< .001
**Reading speed (wpm)**	273.30 (97.11)	236.86 (69.19)	.008
**Total saccadic amplitude (°)**	1269 (551)	4086 (1551)	< .001
**Number of fixations (per word)**	0.80 (0.32)	1.03 (0.32)	< .001
**Fixation duration (ms, on words)**	284.34 (50.52)	228.41 (33.02)	< .001

Regarding the comprehension score, the effect of Media did not reach significance (*F* < 1). We also found that, overall, the participants scored higher on the literal (*M* = 53.52%, *SD* = 23.71) than the inferential questions (*M* = 32.87%, *SD* = 18.91; *F*(1, 39) = 66.67, *p* < .001, *η_p_* ^2^ = 0.631). The interaction between Media and Question Type was not significant (*F* < 1).

To further assess the null effect of Media, we conducted a Bayesian model comparison. The model including Media alone yielded a Bayes Factor of BF₁₀ = 0.18 (±0.81%), providing substantial evidence in favor of the null hypothesis. In contrast, the model including Question Type alone showed decisive evidence for an effect (BF₁₀ = 7,704,209 ± 0.91%). Adding Media to this model (Model 3: Media + Question Type) slightly reduced the Bayes Factor (BF₁₀ = 1,510,999 ± 3.28%), and the full model including the interaction term (Model 4: Media + Question Type + Media × Question Type) yielded a lower Bayes Factor still (BF₁₀ = 330,934 ±1.68%).

Media had a significant effect, showing that comics were explored faster than texts (*F*(1, 39) = 76.602, *p* < .001, *η_p_* ^2^ = 0.663).

Finally, reading speed was higher for comics than for texts (*F*(1, 39) = 6.831, *p* = .013, *η_p_* ^2^ = 0.149).

### 3.2 How do comics help comprehension?

To answer this second question, we conducted two types of analyses. In the first, we tested the hypothesis that the pictorial representation should be faster to generate than the verbal representation when reading comics. We thus performed repeated‐measures ANOVAs with Type of Area of Interest (pictures vs. balloons) as the within variable in comics for **viewing time,** calculated as the total time spent inside the two types of area of interest, as well as the **mean duration and number of fixations**.

For the second type, we tested the efficiency of the extraction of information, when processing the whole page, and the content of the balloons. Thus, for whole‐page processing, we conducted a repeated‐measures anova with Media as the within variable for the **total saccadic amplitude**, calculated as the sum of the amplitudes of all the saccades in comics and text. For the processing of the content of the balloons, we conducted repeated‐measures anovas with Media as the within variable for the **number of fixations** needed to read a word, calculated as the total number of fixations in texts or the number of fixations falling in balloons and captions in comics divided by the total number of words, as well as the mean fixation duration on the words in comics and texts. The descriptive statistics (means and standard deviations) are shown in Table 3.

#### 3.2.2 Viewing time and number and duration of fixations in comic pictures and balloons

Viewing time was shorter in pictures (*M* = 37.79 s, *SD* = 17.47) than balloons (*M* = 99.42 s, *SD* = 44.33, *F*(1, 39) = 90.405, *p* < .001, *η_p_* ^2^ = 0.699). More precisely, the participants made fewer and shorter fixations in pictures (number of fixations: *M* = 153.15, *SD* = 69.71; fixation duration: *M* = 246.87 ms, *SD* = 34.12) than in balloons and captions (number of fixations: *M* = 363.95, *SD* = 198.05; *F*(1, 39) = 60.24, *p* < .001, *η_p_* ^2^ = 0.607; fixation duration: *M* = 284.34 ms, *SD* = 50.52; *F*(1, 39) = 39.494, *p* < .001, *η_p_* ^2^ = 0.503).

#### 3.2.3 Total saccadic amplitude in comics and texts

**Fig. 1:**
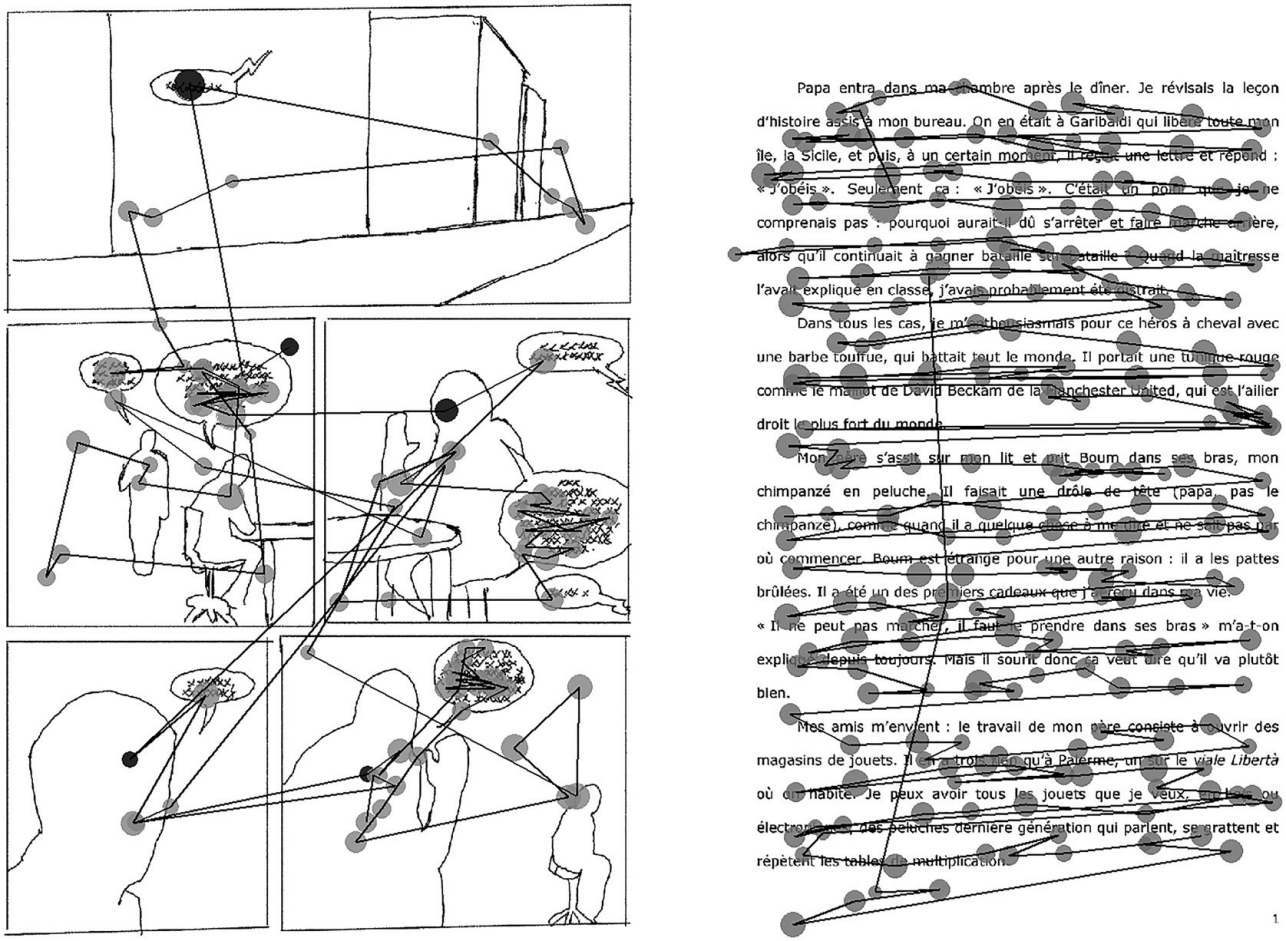
Examples of scanpaths for two participants for the comic (left) and text version of T1. In the comic version, the first fixations of each panel are in a different colors. Circle sizes are proportionate to fixation duration. The comic page has been schematized to comply with copyright policies. The first panel portrays an Italian street scene. Panels two through four depict the main character and his father engaged in a discussion within a bedroom setting. The final panel features the father holding a stuffed monkey in his hand.

Total saccadic amplitude was shorter in comics than in texts (*F*(1, 39) = 136.537, *p* < .001, *η_p_* ^2^ = 0.6; see Fig. 1).

#### 3.2.4 Number and duration of fixations in comic balloons and texts

The words in comics required fewer fixations than those in texts (*F*(1, 39) = 23.73, *p* < .001, *η_p_* ^2^ = 0.378). However, fixations were longer in the comics than in the texts (*F*(1, 39) = 127.03, *p* < .001, *η_p_* ^2^ = 0.765).

Given that reading speed varied with the total number of fixations and fixation duration (Rayner, 1998), we also performed two simple mediation analyses to identify which of these two parameters, number of fixations or fixation duration, mediated the effect of Media (comic book vs. text) on reading speed (Calabrèse, Bernard, Faure, Hoffart, & Castet, 2014). We therefore log‐transformed all three variables and mean‐centered the number of fixations and fixation durations for these analyses. We then conducted a joint significance test (Yzerbyt, Muller, Batailler, & Judd, 2018).

The mediation analyses showed that the total effect of Media on reading speed was significant (*t*(39) = 2.80, *p* = .008). We then tested whether the number of fixations mediated the effect of Media on reading speed. This analysis revealed a significant negative effect of Media on the number of fixations (*t*(39) = 12.63, *p* < .001) and a significant negative effect of the number of fixations on reading speed (*t*(37) = 12.62, *p* < .001). Using the Monte Carlo resampling method, the magnitude of the indirect effect was estimated at 0.574 (95% Confidence Interval CI: [0.451, 0.709]). The effect of Media on reading speed after controlling for the number of fixations was significant but changed direction (*t*(37) = 8.85, *p* < .001). Regarding the effect of Media on reading speed, the results of the mediation analysis of the number of fixations are shown in Table 4. These results suggest the presence of a suppression effect, as the direct and indirect effects have opposite signs (MacKinnon, 2000). In other words, comics reduced the number of fixations, the reduced number of fixations increased reading speed, and this indirect positive effect of Media on Reading Speed through Number of Fixations suppressed the direct negative effect of Media on Reading Speed.

**Table 4:** Mediation analysis pathways between Media, reading Speed, and number of fixations

Path	Estimate	*SE*	*t*	*p*	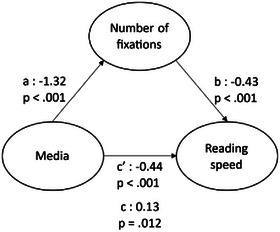
a	−1.32	0.10	*t*(39) = 12.63	< .001***	
b	−0.43	0.03	*t*(37) = 12.63	< .001***	
c	0.13	0.05	*t*(39) = 2.80	.012*	
c’	−0.44	0.05	*t*(37) = 8.69	< .001***	

*Note*. Path a: regression of the Number of Fixations on Media; b: regression of Reading Speed on the Number of Fixations; c: regression of Reading Speed on Media; c’: regression of Reading Speed on Media after controlling for the Number of Fixations.

There was a significant effect of Media on fixation duration (*t*(39) = 12.92, *p* < .001) but no effect of fixation duration on reading speed (*t*(37) = 1.33, *p* = .193). Results from the mediation analysis of the number of fixations on the effect of the Media on reading speed are shown in Table 5. As the fixation duration effect on reading speed was not significant, it is not possible to conclude that the fixation duration effect mediated the effect of Media on reading speed.

**Table 5:** Mediation analysis pathways between Media, Reading Speed, and Fixation Duration

Path	Estimate	*SE*	*t*	*p*	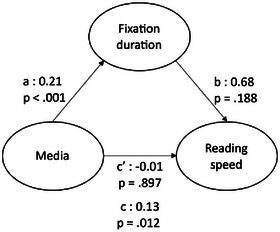
a	0.21	0.02	*t*(39) = 12.92	< .001***	
b	0.68	0.51	*t*(37) = 1.34	.188	
c	0.13	0.05	*t*(39) = 2.62	.012*	
c’	−0.01	0.12	*t*(37) = 0.13	.897	

*Note*. Path a: regression of the number of fixations on Media; b: regression of reading speed on the number of fixations; c: regression of reading speed on Media; c’: regression of reading speed on Media after controlling for fixation duration.

### 3.3 How do we integrate the pictorial and verbal components of comics?

To answer our third question, we conducted a repeated‐measures ANOVA on the results for comics only, with Type of Fixation (first vs. later), and Number of Pass (first visit vs. subsequent visit) as within variables and the **probability of the first and later fixations landing on pictures** as the dependent variable. For this analysis, we used the same procedure as in Laubrock et al.’s (2018) study. Finally, we performed a repeated‐measures anova with Type of Area of Interest (pictures vs. balloons) for the mean **number of runs** as the dependent variable.

#### 3.3.1 Fixation locations

**Fig. 2:**
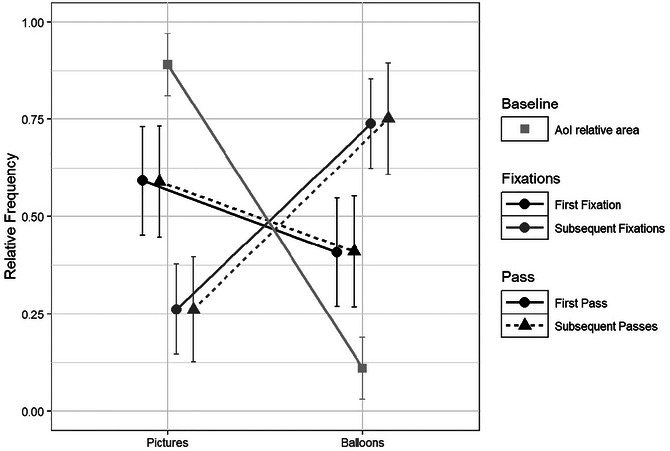
Relative frequency of fixations locations. First fixations are represented in black and subsequent fixations in dark gray. First (circle and full line) and subsequent passes (triangle and dotted line). The relative area occupied by the two components is represented in light gray.

Fig. 2 illustrates the relative frequency of fixations on pictures or balloons as a function of whether or not the fixation was the first fixation of the pass and whether the pass was the first or a subsequent pass. The relative area of the Area of Interest (AoI) is represented in gray for comparison. Pictures represented 88.99% of the area of the panel. When participants visited a panel, their first fixations fell more frequently on pictures (*M* = 0.59, *SD* = 0.14) than was the case for subsequent fixations (*M* = 0.26, *SD* = 0.12; *F*(1,37) = 545.88, *p* < .001, *η_p_* ^2^ = 0.937). There was no effect of the Number of Pass (*F* < 1) and no interaction between Pass and Number of Fixation (*F* < 1).

#### 3.3.2 Number of runs

Pictures (*M* = 2.30, *SD* = 0.78) received more runs than balloons and captions (*M* = 1.99, *SD* = 0.67; *F*(1, 39) = 39.237, *p* < .001, *η_p_* ^2^ = 0.502).

## 4 Discussion

The main aim of this study was to better understand the cognitive processes involved in the potential benefits for comprehension of comics, compared to traditional text. We formulated three questions, based on two models: the cognitive theory of multimedia learning (CTML; Mayer, 2009) and Sonine and Chanquoy's (2007) model.

### 4.1 Do comics facilitate the elaboration of the representation of the content of the story?

As previous studies have presented many methodological biases, we first asked this very general question in order to determine if comics can facilitate the building of the final representation of the story and the processing of the verbal component more than is the case with traditional texts. Regarding the final representation, the CTML predicts that comics will result in better comprehension than text as comics display both verbal and pictorial elements. Consistently with previous studies showing similar comprehension performances across text and visual narratives (Baggett, 1979; Kendeou et al., 2008), we observed no differences in reading comprehension between comics and traditional texts either for literal or inferential questions. This null effect is unlikely to reflect a measurement issue: participants performed significantly better on literal than on inferential questions, confirming that the comprehension questions were sensitive and discriminative. A Bayesian analysis further supported the absence of a format effect, providing substantial evidence in favor of the null hypothesis. The quality of the final representation of the story may therefore be similar for the two media. By contrast, we did find shorter exploration times for comics than for traditional texts. Comics might have allowed readers to extract as much information as texts but within a shorter amount of time.

The results of previous studies concerning the multimedia effect on comprehension are inconsistent, with some studies showing that skilled adult readers understand comics better than texts (Aleixo & Sumner, 2017; Wong et al., 2017) and others finding no differences between comics and texts (Nalu & Bliss, 2011; Short et al., 2013; Yum et al., 2021). We have argued that methodological differences, such as the procedure used to create the comic or text supports or the use of a time constraint, could explain this lack of consistency. In the field of scientific communication, Farinella (2018) suggested that the benefit of comics for comprehension and memory may be due to the introduction of a narrative structure, with the characters and storytelling encouraging engagement on the part of readers and their identification with the characters. In his PINS model, Cohn (2020b) argues that pictures not only carry semantic information, which is processed to build a situation model of the story being read but also contain a narrative structure essential for comprehension. According to this model, narrative and semantic processing occur in parallel and interact to facilitate story comprehension. Thus, the presence of a narrative structure may better explain the benefits of comics for comprehension than the comic format itself. By removing the pictorial component, authors such as Aleixo and Sumner (2017) may have eliminated not only important semantic information from their comic material but also the benefits that the narrative form created by characters and storytelling may provide for comprehension.

Time constraints during reading task may also have influence previous results. For instance, in Wong et al.’s (2017) study, comics were adapted from texts, with text passages being converted into pictures where appropriate. To control for reading time, the authors applied the same time constraint for texts and comics (8 min). However, as reported in our study, comics are processed faster than traditional texts. A time constraint of 8 min may therefore be longer than is needed to explore the entire comic, allowing readers to read the same passage several times and elaborate exploration strategies, but too short for them to be able to elaborate the same kinds of strategies when reading traditional texts. In our study, we controlled for the presence of a narrative and allowed readers to explore the supports at their own speed and did not observe that comics facilitated comprehension any more than traditional texts.

These results question the interpretation put forward by the CTML to explain the *multimedia effect* (Mayer, 2009), whereby the presence of a picture is thought to enhance comprehension as it encourages readers to create a pictorial mental model of the content of the supports and to make connections between the verbal and the pictorial model. Even though comics fulfill many of the criteria described in the CTML as fostering comprehension and learning, such as the spatial contiguity principle, which implies that supports are better learned when words and pictures are presented near each other, the presence of pictures is not sufficient to induce better comprehension scores when reading comics, compared with traditional texts. Finally, few studies have highlighted the ability of comics to reduce the time needed to acquire information (but see Nalu & Bliss, 2011). Here, we showed that comics may be an interesting tool in contexts where readers need to process information in a limited time frame.

Regarding the effect of comics on the processing of the verbal component, the faster reading speed revealed that words were read more efficiently in comic balloons than in traditional texts, a finding that is consistent with Sonine and Chanquoy's (2007) model. This suggests that the presence of pictures does indeed facilitate the processing of the words in balloons and captions. These results are particularly important as ours is the first study to show that it can be easier to read information in comics than in traditional texts. Faster reading speed has also been found in a multimedia learning context, where seeing a picture for as little as 600 ms before reading the associated text significantly reduced reading time, compared with reading the same text without pictures (Eitel, Scheiter, & Schüler, 2013). To explain their results, Eitel and colleagues developed an explanation that complements that proposed by Sonine and Chanquoy. This takes the form of the Scaffolding view hypothesis, which assumes that spatial information is better represented and more easily extracted from pictures. Thus, when processing a multimedia text about a pulley system, viewing the picture first may help readers to build a mental model of the global spatial structure of the system and prevent them from building an inadequate representation when reading the text.

### 4.2 How do comics help comprehension?

To answer this question, we first tested the central assumption of Sonine and Chanquoy's model (2007), according to which pictures are easier and faster to process than words in comics. We observed fewer and shorter fixations, resulting in shorter viewing times in comic pictures than in balloons. It thus seems to be easier to elaborate the pictorial than the verbal representation. Although this result is not consistent with the scene perception literature, which typically shows longer fixation durations when viewing images than when reading words (Rayner, 2009; Rayner, Rotello, Stewart, Keir, & Duffy, 2001; Underwood, Hubbard, & Wilkinson, 1990), this pattern has been found in several studies using comics with adults (Laubrock et al., 2018) and sixth graders (Kinzer et al., 2012). Laubrock et al. (2018) argued that this result may come from the fact that visual scenes depicted in comics are less complex than natural scene perception, thus leading to shorter fixations. Second, in accordance with our hypothesis, we observed shorter total saccadic amplitude in comics than in traditional texts, reflecting the fact that it is easier to extract information from comics than texts (Kotval & Goldberg, 1998). Since part of the information in comics is pictorial, and because the verbal component is embedded in the pictures via balloons and captions, information that is important for comprehension may be more salient and easier to retrieve in comics than in text. This can be seen particularly clearly in Fig. 1, which shows the scanpath of two participants reading the text and comic versions of the same story. This, in turn, could reduce the time needed to explore the support.

Regarding the oculomotor behavior involved in elaborating the verbal representation, we expected that the faster reading speed would be explained by shorter and fewer fixations on comic balloons than on traditional texts. This hypothesis was partially confirmed, as we observed fewer, but longer, fixations on words in balloons than in traditional texts. This is an interesting finding since fewer and shorter fixations may commonly be associated with easier, less demanding processing (Krejtz, Duchowski, Krejtz, Kopacz, & Chrząstowski‐Wachtel, 2016; Rayner et al., 2006). Several hypotheses can explain why we observed longer fixations on the balloons in comics. First, it could be argued that longer fixations mean that balloons in comics are processed more deeply than texts in novels (Alemdag & Cagiltay, 2018). For instance, Jakobsen and Jensen (2008) showed that reading while translating elicited a longer mean fixation duration than reading for understanding. Another interpretation is that longer fixations may be due to the elicitation of a picture/text integration process that begins while the verbal component is still being processed as suggested by Sonine and Chanquoy's (2007) model. Longer fixations could also be due to the different fonts used in comics (Franken, Podlesek, & Možina, 2015; Josephson, 2008). Indeed, because we used already‐published comics, we did not control for the fonts used in these and in traditional texts. Thus, the fonts in the comics had a handwritten style, which is typical of comics and could result in a longer mean fixation duration. Although mean fixation durations on words are longer in comics, the mediation analysis showed that their effect on reading speed is suppressed by the reduction of the number of fixations. In other words, the increase in fixation duration in comics did not slow down reading speed, as the decreased number of fixations may have compensated for this effect. This finding is consistent with previous studies showing that reading speed is mainly influenced by the number of fixations rather than by fixation duration (Calabrèse et al., 2014).

### 4.3 How do we integrate pictorial and verbal components of comics?

As predicted by Sonine and Chanquoy's (2007) model, which expects pictorial information to be given priority when building the representation of the content of the story, we observed that when readers entered a panel, their first fixations tended to land on pictures more often than subsequent fixations, suggesting that priority may be given to the processing of the pictures in order to build a first basic representation of the content of the panel. The effect was similar in both first and subsequent passes, suggesting that readers specifically target the picture first when processing a panel. Similar results have been reported in Laubrock et al. (2018), who analyzed a corpus of six comic book excerpts, amounting to a total of 108 pages, read by 100 adult skilled readers, as well as for single‐frame cartoons (Carroll et al., 1992).

According to Sonine and Chanquoy (2007), processing the picture first allows readers to extract the semantic information from it and create a first representation of the content of the panel. They could then use this pictorial semantic information to implement top‐down processes that may facilitate the processing of the verbal component, thus reducing the number of fixations needed to process the words in balloons and enhance reading speed. One might argue that this interpretation holds if reading speed increases only in panels in which pictures are fixated before the words in balloons. However, even when balloons are fixated first, some evidence suggests that pictures processing may occur in parafoveal vision, during the word reading within balloons (Laubrock et al., 2018). Thus, a facilitation effect for word reading may be observable even if the picture is not fixated first.

Our results align with the SPECT (Loschky et al., 2020). This model distinguishes between front‐end processing, which enables the extraction of picture content, and back‐end processing, which is involved in constructing an event model when viewing a story with pictures only. Information derived from front‐end processing feeds into back‐end processing to develop a representation of the story's content. Attentional selection, a front‐end process, may be influenced by top‐down attentional control from the event model or relevant world knowledge.

Regarding the number of runs in pictures and balloons, and still in line with Sonine and Chanquoy's hypotheses concerning the role of pictures in the building of the basic representation, our results showed that pictures were revisited more often. This may reflect the integration of the verbal and pictorial components of the panel, with participants going back to the pictures after reading the content of the balloons or captions in order to correct or confirm the content of the semantic representation of the text being processed (Carroll et al., 1992; Rayner et al., 2001).

### 4.4 Limitations and future directions

One limitation of this study is its lack of statistical power, as we observed no differences between reading formats and no interaction between the Media and Question Type variables with regards to reading comprehension scores. This may have been due to the small sample size. Results of a post hoc power analysis using the R package Superpower (Lakens & Caldwell, 2021) and based on the effect size of the reading comprehension score indicated that, in order to obtain 80% power, 2271 participants would have been needed to show a significant difference between comics and texts and 1859 to show a significant interaction between Media and Question Type. However, we may argue that the very high sample size needed highlights the fact that the difference between comics and texts for reading comprehension may be negligible.

Future studies will need to address several questions that we could not answer in the present study. For example, Kirtley et al. (2018) demonstrated that certain panel characteristics, such as balloon size and position, can influence information extraction. It would therefore be valuable to investigate how these characteristics affect attentional selection and mental model construction.

As Cohn (2016) pointed out, linguistic and graphic content can play distinct roles in narrative construction, either reinforcing the same information or conveying complementary elements. Depending on the structure, graphic narratives may be image‐dominant or text‐dominant. Our study does not disentangle these different configurations. Future research should investigate how the relative dominance and informational overlap between images and text modulate the integration processes involved in multimodal narrative comprehension.

Our findings also relate to the broader modularity versus interactivity debate in psycholinguistics. While classical models suggest that lexical and syntactic processing occur in an encapsulated, bottom‐up manner (Fodor, 1983; Swinney, 1979), more recent interactive accounts have shown that contextual and visual information can influence language processing from early stages (Altmann & Kamide, 1999; Tanenhaus, Spivey‐Knowlton, Eberhard, & Sedivy, 1995). Although our results support the idea that pictorial information facilitates the processing of verbal content in comics, they do not enable us to reach a conclusive decision as to the stage at which this facilitation occurs. Future research should explore whether the observed effects arise during lexical access, syntactic parsing, or later semantic integration.

### 4.5 Conclusion

Taken together, these results support the hypothesis that reading comprehension in comics relies critically on the processing of images. Readers first construct a representation of the story's content based on the pictures, which is then refined or corrected with the processing of words in the balloons and captions, using the information provided by the images.

As pictures in comics are faster and easier to process and carry semantic information that would otherwise be contained in text form, comics can be processed and understood faster. Although the CTML is the reference model in multimedia research, our results highlight several limitations of this model when studying comics. As discussed earlier, and contrary to the predictions of the CTML, we did not find any improvement in reading comprehension when comparing comics to traditional texts, suggesting that the presence of pictures is not enough to enhance comprehension. In addition, the CTML is based on the assumption that there are two processing systems. The first of these is pictorial and is dedicated to perceptual information such as color or shape. The second is verbal and processes abstract semantic information (dual coding theory; Paivio, 1986). This assumption implies that the semantic processing of words should be faster than that of pictures, as words are directly stored as abstract semantic concepts. However, our own results as well as previous studies (Potter & Faulconer, 1975; Shaul & Rom, 2019) show the opposite pattern of results, with the semantic processing of pictures being faster than that of words in balloons. This faster semantic processing of pictures may be responsible for the faster reading speed observed in comic‐book balloons, compared to traditional texts. Consequently, the CTML does not seem appropriate for exploring reading processes in comics. As an alternative model, Sonine and Chanquoy's proposal provides a better understanding of the cognitive processes reflected by our results by considering the top‐down influence of pictorial semantic information on text processing. However, future studies will have to further explore the causal relationship between the presence of pictures and faster reading speed in comics.

## Conflict of Interest Statement

The authors declare no conflicts of interest.

## Data Availability

The authors did not receive permission to reproduce the material used in the study. The data that support the findings of this study are openly available in the OSF repository at https://osf.io/usm3x/?view_only=6050a582019c4407b1ddb1fa4fb672e1. The materials used in this study are not publicly available due to copyright restrictions.
